# Subcutaneous Sarilumab in hospitalised patients with moderate-severe COVID-19 infection compared to the standard of care (SARCOVID): a structured summary of a study protocol for a randomised controlled trial

**DOI:** 10.1186/s13063-020-04588-5

**Published:** 2020-09-09

**Authors:** Rosario Garcia-Vicuña, Francisco Abad-Santos, Isidoro González-Alvaro, Francisco Ramos-Lima, Jesús Sanz Sanz

**Affiliations:** 1 Rheumatology Department, Hospital Universitario de la Princesa, Madrid, Spain; 2 Faculty of Medicine, Universidad Autónoma de Madrid (UAM), Madrid, Spain; 3 Instituto de Investigación Sanitaria La Princesa (IIS-IP), Madrid, Spain; 4 Clinical Pharmacology Department, Unidad de Investigación Clínica y Ensayos Clínicos (UICEC), Hospital Universitario de la Princesa, Plataforma SCReN (Spanish Clinical Research Network), Madrid, Spain; 5 Medical Affairs Department, Immunology Area, Sanofi Spain, Madrid, Spain; 6 Division of Infectious Diseases, Internal Medicine Department, Hospital Universitario de la Princesa, Madrid, Spain

**Keywords:** COVID-19, Randomised controlled trial, Open label trial, protocol, SARS-CoV-2, Coronavirus infections, Sarilumab, IL-6 receptor inhibitors

## Abstract

**Objectives:**

The main aim of the study is to evaluate the efficacy of a single dose of sarilumab, in subcutaneous administration, in hospitalised patients with moderate to early severe COVID-19 infection compared to the current standard of care, to prevent progression to systemic hyperinflammatory status. Our hypothesis is that use of subcutaneous sarilumab in early stages (window of opportunity) of COVID-19 moderate-severe pneumonia can prevent higher oxygenation requirements through non-invasive and invasive mechanical ventilation and decrease in-hospital stays, as well as death rate.

The secondary objectives of the study are to evaluate the safety of sarilumab through hospitalisation and up to day 14 after discharge, compared to the control arm as assessed by incidence of serious and non serious adverse events (SAEs).

In addition, as an exploratory objective, to compare the baseline clinical and biological parameters, including serum IL-6 levels, of the intervention population against controls of the same pandemic outbreak (using a propensity score) to search for markers that identify the best candidates for the treatment with subcutaneous IL-6R inhibitors and to attempt an approximation in the temporal frame of the “window of opportunity”

**Trial design:**

SARCOVID is an investigator-initiated single center randomised proof of concept study.

**Participants:**

Patients treated at the Hospital Universitario La Princesa, Madrid, Spain requiring hospitalisation will be consecutively recruited, meeting all inclusion criteria and none of the exclusion criteria

Inclusion criteria

a. Age >18, <80 years old

b. COVID-19 infection documented by a positive RT-PCR test or, in absence of a RT-PCR positive test, case definition of COVID 19 infection/pneumonia as per local protocol and the presence of a positive serologic test (IgM/IgA by ELISA)

c. Documented interstitial pneumonia requiring admission and at least two of the following parameters:

1) Fever ≥ 37.8°C (tympanic)

2) IL-6 in serum ≥ 25 pg/mL (in the absence of a previous dose of prednisone or equivalent> 1 mg / kg) or PCR> 5mg/dL

3) Lymphocytes <600 cells/mm^3^

4) Ferritin> 300 μg/L that doubles in 24 hours

5) Ferritin> 600 μg/L in the first determination and LDH> 250 U/L

6) D-dimer (> 1 mg/L)

d. Informed verbal consent or requested under urgent conditions, documented in the electronic medical record.

Exclusion criteria

a. Patients who require mechanical ventilation at the time of inclusion.

b. AST / ALT values > 5 folds the ULN.

c. Absolute neutrophil count below 500 cells/mm^3^

d. Absolute platelet count below 50,000 cells/mm^3^

e. Documented sepsis or high suspicion of superimposed infection by pathogens other than COVID-19.

f. Presence of comorbidities that can likely lead to an unfavourable result according to clinical judgment.

g. Complicated diverticulitis or intestinal perforation.

h. Current skin infection (eg, uncontrolled dermopiodermitis).

i. Immunosuppressive anti-rejection therapy.

j. Pregnancy or lactation.

k. Previous treatment with tocilizumab or sarilumab.

l. Patients participating in another clinical trial for SARS-CoV-2 infection.

m. Patients with known hypersensitivity or contraindication to sarilumab or excipients.

**Intervention and comparator:**

The intervention group, sarilumab plus standard of care, will receive 400 mg single dose treatment with Sarilumab (Kevzara), 2 subcutaneous injections 200mg each in a pre-filled syringe. Treatment with drugs or procedures in routine clinical practice that the clinician responsible for the patient deems necessary is allowed.

The control group will receive drugs or procedures in routine clinical practice according to the best standard of care as per local protocol.

**Main outcomes:**

Primary Outcome Measures

1. Mean change in clinical status assessment using the 7-point ordinal scale at day 7 after randomisation compared to baseline (Score ranges 1-7)

1. Death;

2. Hospitalised, requiring invasive mechanical ventilation or extracorporeal membrane oxygenation (ECMO);

3. Hospitalised, requiring non-invasive ventilation or high flow oxygen devices;

4. Hospitalised, requiring supplemental oxygen;

5. Hospitalised, not requiring supplemental oxygen - but in need of ongoing medical care (COVID-19 related or otherwise)

6. Hospitalised, not requiring supplemental oxygen - no longer requires ongoing medical care (independent)

7. Not hospitalised

2. Duration of hospitalisation: Days from the date of enrolment to the date of discharge

3. Number of deaths at the end of study

**Randomisation:**

Randomisation to treatment arms sarilumab plus standard of care or standard of care in a 2:1 ratio will be performed by the Clinical Research and Clinical Trials Unit (CRCTU) at the Hospital using a table of random numbers, an internet-based randomisation tool. After checking that all inclusion criteria are met and none of the exclusion criteria, CRCTU will communicate the recruiting investigator the assigned treatment.

**Blinding (masking):**

This study is unblinded.

**Numbers to be randomised (sample size):**

30 patients treated by COVID-19 infection who require hospitalisation: 20 will receive sarilumab plus Standard of Care and 10 will receive Standard of Care.

**Trial Status:**

The Protocol version number is 2, as of 6^th^ April 2020, with amendment 1, as of 7^th^ May 2020. The recruitment is ongoing. Recruitment started on April 13^th^ 2020 and is anticipated to be completed by November 2020.

**Trial registration:**

This trial was first registered in the European Union Clinical Trials Register on 4 April 2020, EudraCT Number 2020-001634-36. Then, posted on ClinicalTrials.gov on 22 April 2020, Identifier: NCT04357808.

**Full protocol:**

The full protocol is attached as an additional file, accessible from the Trials website (Additional file 1). In the interest in expediting dissemination of this material, the familiar formatting has been eliminated; this Letter serves as a summary of the key elements of the full protocol.

The study protocol has been reported in accordance with the International Council Harmonization guidelines*:* *https://www.ich.org/page/efficacy-guidelines*.

## Supplementary information

**Additional file 1.** Full Study Protocol.

## Data Availability

RGV, FAS and IGA will have access to the final trial dataset. The data will be available from the author on reasonable request. Contact details: Rosario García de Vicuña. mariadelrosario.garcia@salud.madrid.org Rheumatology Unit, Hospital Universitario de la Princesa, IIS-IP, Diego de León 62, 28006, Madrid Until publication, only research staff have access to the scientific and personal data, in agreement with Regulation (EU) 2016/679, of the European Parliament and of the Council, of 27th April 2016.

