# Supplementary material for: Subcutaneous Sarilumab in hospitalised patients with moderate-severe COVID-19 infection compared to the standard of care (SARCOVID): a structured summary of a study protocol for a randomised controlled trial
Source: Trials. 2020 Sep 9;21:772. doi: 10.1186/s13063-020-04588-5 (PMC7480631; doi:10.1186/s13063-020-04588-5)
Supplement: Supplementary file 1 — Additional file 1. Full Study Protocol. [file 13063_2020_4588_MOESM1_ESM.pdf]

CLINICAL TRIAL PROTOCOL

Title: "RANDOMIZED, OPEN LABEL, PROOF OF CONCEPT STUDY TO EVALUATE THE EFFICACY OF SUBCUTANEOUS SARILUMAB IN PATIENTS WITH MODERATE-SEVERE COVID-19 INFECTION "

Protocol Code:	SARCOVID
Version:	2.0; April, 6th, 2020
Amendment 1:	May 7th, 2020
EudraCT No.:	2020-001634-36

Principal Investigator: Rosario García de Vicuña
Rheumatology Service
University Hospital La Princesa.

Coordinator: Jesús Sanz Sanz
Infectious Internal Medicine Service
University Hospital La Princesa

Sponsor: Rosario García de Vicuña
Rheumatology Service
University Hospital La Princesa

STRICTLY CONFIDENTIAL: Property of Hospital Universitario de la Princesa.
It is not allowed the dissemination or replication out of terms of this proposal

Protocol Signature Page

Tittle: "RANDOMIZED, OPEN LABEL, PROOF OF CONCEPT STUDY TO EVALUATE THE EFFICACY OF SUBCUTANEOUS SARILUMAB IN PATIENTS WITH MODERATE-SEVERE COVID-19 INFECTION"

Protocol Code:	SARCOVID
Version:	2.0; 6th April 2020
Amendment 1	7th May 2020
Nº EudraCT:	2020-001634-36

The undersigned confirm that they agree to the protocol and all the referenced documents, and agree to carry out the study under the conditions described in this protocol and in accordance with the Standards of Good Clinical Practice and the mandatory regulatory requirements.

7th May 2020

________________________________ ________________________________

Rosario García de Vicuña — Date
Sponsor and Principal Investigator
University Hospital La Princesa

7th May 2020

________________________________ ________________________________

Jesús Sanz Sanz — Date
Coordinator Investigator
University Hospital La Princesa

STRICTLY CONFIDENTIAL: Property of Hospital Universitario de la Princesa.
It is not allowed the dissemination or replication out of terms of this proposal

Index

Protocol Signature Page 2

Index 3

List of abbreviations 5

1. Study summary 6

2. Justification of the Study 8
- 2.1 Introduction 8
- 2.2 Description and rationale for the route of administration, dose, guideline and treatment period 13
- 2.3 Study population 13
- 2.4 Declaration that the trial will be conducted in accordance with the protocol, the GCP and the relevant legal requirements 13

3. Study hypothesis 14
- 3.1 Study objectives 14

4. Clinical Study design 14
- 4.1 Study Design 14
- 4.2 Study Endpoints 15
- 4.3 Randomization process 15
- 4.4 Masking process 15
- 4.5 Treatment period 15
- 4.6 Schedule of clinical trial evaluations 15
- 4.7 Data Management 16
- Definition of end of Study 16

5. Study population 16
- 5.1 Inclusion criteria 16
- 5.2 Exclusion criteria 16
- 5.3 Withdrawal and analysis criteria 17

6. Patients treatment 17
- 6.1 Definition of treatment 17
- 6.2 Procedure to monitor subject compliance 17
- 6.3 Interaction with other medicines and other forms of interaction 17

7. Study development 17
- 7.1 Study visits 17
- 7.2 Study Calendar and Procedures 18
- 7.3 Quality Control - Monitoring 18
- 7.4 Collecting clinical data 19

8. Efficacy measures 19

9. Safety measures. 20
- 9.1 Definitions 20
- 9.2 Security Reference Information 21

STRICTLY CONFIDENTIAL: Property of Hospital Universitario de la Princesa.
It is not allowed the dissemination or replication out of terms of this proposal

9.3 Collecting adverse events/pregnancies 21
9.4 SAE that do not require notification to the Pharmacovigilance Unit 22
9.5 Procedure for notification of SAE/pregnancies to the Sponsor 22
9.6 Expedited notification of SUAR to Health Authorities/CEIm 23
9.7 Notification deadlines 23
9.8 Expedited notification of other relevant security information 23
9.9 Annual safety report 23
9.10 Notification to investigators 24
9.11 General and particular standards for researchers 24

10. Statistical analysis 24
10.1 Data management 24
10.2 Description of statistical methods 24
10.3 Sample Size 25

11. Direct access to data/source documents 25

12. Quality control and assurance 26
12.1 Deviations from protocol 26

13. Ethics 26
13.1 General and particular standards for researchers 26
13.2 Ethics Committees for investigation with medicinal products (CEIm) 27
13.3 Informed consent 27
13.4 Amendments to the protocol 27
13.5 Security and confidentiality devices 27

14. Handling data and archiving records 27
14.1 Documentation Archive 27
14.2 Case report form (CRF) / data record 28
14.3 Medication identification 28

15. Insurance 29

16. Publishing policy 29

References 30

APPENDIX A: Declaración de Helsinki de la AMM 31

APPENDIX B: Serious and Unexpected Adverse Reactions Notification Form (SUARs Form) (Spanish) 36

APPENDIX C: authorization from the Spanish Agency for Medicines and Healthcare Products (AEMPS) and the favourable opinion of a Committee on Ethics of Research with Medicines. ..... ¡Error! Marcador no definido.

List of abbreviations

AE	Adverse event
SAE	Severe Adverse event
AEMPS	Agencia Española de Medicamentos y Productos Sanitarios
RA	Rheumatoid Arthritis
GCP	Good Clinical Practices
CAR-T	Chimeric Antigen Receptors – T cell
CEIm	Ethics Committees for investigation with medicinal products
IC	Informed consent
CRF	Case report form
ICH	International Conference on Harmonisation
IL-6	Interleukin 6
CRP	C Reactive Protein
AR	Adverse Reaction
SAR	Severe Adverse Reaction
SUAR	Severe and Unexpected Adverse Reaction
SAR	Sarilumab
SARS	Systemic Acute Respiratory Syndrome
SC	Subcutaneous
CRS	Cytokine Release Syndrome
TCZ	Tocilizumab

STRICTLY CONFIDENTIAL: Property of Hospital Universitario de la Princesa.
It is not allowed the dissemination or replication out of terms of this proposal

1. Study summary

1. Type of application

Clinical trial with low-level of intervention.

2. Sponsor

Rosario García de Vicuña
Rheumatology Service.
University Hospital La Princesa.
Diego de León 62, 28006 Madrid
Teléfono: +34 91 5202473
Email: mariadelrosario.garcia@salud.madrid.org

3. Tittle

RANDOMIZED, OPEN LABEL, PROOF OF CONCEPT STUDY TO EVALUATE THE EFFICACY OF SUBCUTANEOUS SARILUMAB IN PATIENTS WITH MODERATE-SEVERE COVID-19 INFECTION.

4. PROTOCOL ID

Protocol Code:	SARCOVID
Version:	2.0, April 6th 2020
Nº EudraCT:	2020-001634-36
Amendment 1:	7th May 2020

5. Principal Investigator

Rosario García de Vicuña
Rheumatology Service.
University Hospital La Princesa
Address: Diego de León, 62, 28006 - Madrid
Telephone: +34 91 5202473
Email: mariadelrosario.garcia@salud.madrid.org

6. Coordinator Investigator:

Jesús Sanz Sanz
Infectious Internal Medicine Service
University Hospital La Princesa
Address: Diego de León, 62, 28006 – Madrid
Telephone: +34 915204014
Email: jsanzsanz@salud.madrid.org

7. Collaborating Investigators

Ángela Gutiérrez, Internal medicine/Infectious diseases Service
José Curbelo, Internal medicine/Infectious diseases Service
Natalia Villalba, Emergency Service.
Andrés von Wernitz, Emergency Service.
Tamara Alonso Pérez, Pneumology Service.
Pedro Landete, Pneumology Service.
Irene Llorente Cubas, Rheumatology Service.
Eva Tomero Muriel, Rheumatology Service

STRICTLY CONFIDENTIAL: Property of Hospital Universitario de la Princesa.
It is not allowed the dissemination or replication out of terms of this proposal

Sebastián Rodriguez García, Rheumatology Service.
Esther Ramírez Herráiz, Hospital Pharmacy Service.
Laura Cardeñoso Domingo, Microbiology Servie
Aránzazu Alfranca, Immunology Servie.
Isidoro González Álvaro, Rheumatology Service
María Ángeles Sanz de Benito, Clinical Laboratory Service.
Francisco Abad Santos, Clinical Pharmacology Service.
Gina Mejia Abril, Clinical Pharmacology Service.

8. Ethics Committees for investigation with medicinal products (CEIm)

CEIm in University Hospital La Princesa.

9. Name and rating of the person responsible for monitoring

Clinical Research and Clinical Trials Unit
Clinical Pharmacology Service
University Hospital La Princesa
Address: Diego de León, 62
28006 - Madrid
Telephone: +34 91 5202540

10. Name of pharmaceutical laboratories or other institutions involved in the study

Sanofi (provides medication and funds for a data manager, in agreement with the achievement of predefined milestones throughout the study)

11. Research drug

Sarilumab (Kevzara®).

12. Clinical trial phase

Phase II clinical trial.

13. Objectives

- To assess the efficacy of early administration of sarilumab subcutaneously in patients with moderate-severe early-stage COVID-19 infection, in comparison to the current standard of care.
- The secondary objectives of the study are to evaluate the safety of sarilumab through hospitalization and up to day 14 after discharge, compared to the control arm as assessed by incidence of serious and non serious adverse events (SAEs).
- In addition, as an exploratory objective, to compare the baseline clinical and biological parameters, including serum IL-6 levels, of the intervention population against controls of the same pandemic outbreak (using a propensity score) to search for markers that identify the best candidates for the treatment with subcutaneous IL-6R inhibitors and to attempt an approximation in the temporal frame of the “window of opportunity”

14. Primary Outcomes

- Mean change in clinical status assessed by the 7-point ordinal scale from study inclusion to day 7 (after randomization)

  1. Death;
  2. Hospitalized, requiring invasive mechanical ventilation or extracorporeal membrane oxygenation (ECMO);

3. Hospitalized, requiring non-invasive ventilation or high flow oxygen devices;
4. Hospitalized, requiring supplemental oxygen;
5. Hospitalized, not requiring supplemental oxygen - requiring ongoing medical care (COVID-19 related or otherwise)
6. Hospitalized, not requiring supplemental oxygen - no longer requires ongoing medical care (independent)
7. Non-hospitalized

- Duration of hospitalization (days)
- Mortality rate at the end of the study.

15. Design

Randomized clinical trial, open-label, one site, sarilumab + Standard of Care vs Standard of Care, in a ratio 2:1.

16. Disease under study

COVID-19 infection that needs hospitalization.

17. Study population and total number of patients

30 patients treated by COVID-19 infection who require hospitalization: 20 will receive sarilumab plus Standard of Care and 10 will receive Standard of Care.

18. Study treatment

Kevzara 200 mg, 2 Injections SC, in pre-filled syringe, single dose. Total amount 400 mg.

19. Duration of treatment

Single dose in the first visit.

20. Schedule and expected date of completion

The overall duration of the study is estimated at about 2 months, from patient selection to the last scan. The rate of recruitment may vary depending on the situation of the pandemic and the possible referral of moderate cases to other centers.

2. Justification of the Study

2.1 Introduction

The global health emergency created by the rapid spread of SARS-CoV-2 coronavirus poses a particularly difficult challenge for health services, of which there is no precedent of this magnitude. With the commitment of all sectors, the action of the health system is critical to prevent its transmission in the community and the early detection of COVID-19 disease, in order to establish all the necessary pharmacological and non-pharmacological interventions, adjusted to the levels of risk. Therapeutic decisions must be taken urgently in a rapidly changing scenario with a high degree of uncertainty. At this point, clinical research is critical to defining which new drugs or new uses of already authorized drugs are effective and safe for COVID-19.

This proposal corresponds to a proof-of-concept study that aims, in a short period of time, to answer the question of whether sarilumab, an IL-6 receptor inhibitor, in subcutaneous

formulation, may be effective in the treatment of moderate-severe COVID-19 infection in early stages (window of opportunity) to prevent progression to the state of systemic hyper inflammation, with serious pro-forecast implications.

2.1.1 IL-6 Receptor Blockers

TOCILIZUMAB

It is a human monoclonal antibody that blocks IL-6 receptor that is approved for the treatment of rheumatoid arthritis (RA) on the front line and in failure to anti-TNF, Juvenile Idiopathic Arthritis (JIA), Giant Cell Arteritis (GCA) and severe Cytokine Release Syndrome (CRS) secondary to CAR-T cells therapy. https://www.ema.europa.eu/en/documents/product-information/roactemra-epar-product-information_es.pdf

Tocilizumab is available for IV administration (80 and 200 mg vials) and in pre-filled syringes for SC administration of 162 mg. The usual dose in AR, JIA and ACG is 4-8 mg/kg in monthly infusions. It also has indication in the CRS by CAR, in which higher doses (up to 8-12 mg/kg) are recommended that can be repeated up to every 8 hours, without exceeding 800 mg per dose.

No adjustment is required in renal impairment patients. Its safety is not established in patients with liver disease.

SARILUMAB

Sarilumab is a human monoclonal antibody that binds membrane-bound and soluble IL-6 receptors to inhibit IL-6 signalling, licensed for the treatment of RA in a subcutaneous route administration. Several clinical trials are now underway in GCA and polymyalgia rheumatica (PMR). https://cima.aemps.es/cima/dochtml/ft/1171196001/FT_1171196001.html

https://www.ema.europa.eu/en/documents/product-information/kevzara-epar-product-information_es.pdf

Kevzara® (Sarilumab, SAR) is available in syringes and auto-injectors of 200 and 150 mg. The dose regimen for rheumatoid arthritis is 200 mg every 2 weeks.

Its affinity for the IL-6 receptor is 20-fold higher than tocilizumab, has a good absorption after single s.c. administration, with a bioavailability about 80% of the amount administered. The maximum plasma concentration (t_{max}) is reached within 2 to 4 days, independently of the dose used. The initial half-life of sarilumab is 8 to 10 days (after single dose). The approved dosing schedule for treatment with sarilumab in AR is one s.c. injection every 2 weeks, with which stationary concentrations of sarilumab are obtained within 12 to 16 weeks after the start of treatment, with a build-up of 2 to 3 times, compared to a single dose exposure. When the plasma levels of sarilumab are high, their elimination depends on that concentration, as the linear type elimination mechanisms predominate. For this reason, the effective half-life of sarilumab extends to 21 days when the stationary plasma levels of this antibody are reached.

The pharmacodynamic effect and the safety of a single dose of sarilumab s.c (150 and 200 mg) and tocilizumab i.v. (4 and 8 mg/kg) were evaluated in two open, randomized, Japanese-population (n-30) (PDY14191 [NCT02404558]) and non-Japanese (n-101) (6R88-RA-1309 [NCT02097524]) of RA patients) (1). Although there were differences in baseline

pharmacodynamic parameters between the two studies, the onset of the effect on absolute neutrophil count, C-reactive protein (PCR), serum IL-6 and IL-6 soluble receptor levels, during the first week after a single dose, was similar, regardless of the drug, dose or route of administration. The maximum effects on the nadir of neutrophil count and PCR, and on serum IL-6 peaks and IL-6 soluble receptor were comparable in both studies for sarilumab and tocilizumab. The pharmacodynamic response was longer for tocilizumab i.v. and there were no substantial differences in safety. The attached table of the original publication shows changes from the baseline of the main pharmacodynamic and safety variables.

	6R88-RA-1309 (non-Japanese patients[a])				PDY14191 (Japanese patients)	
	SC Sarilumab 150 mg + MTX (N=26)	SC Sarilumab 200 mg + MTX (N=26)	IV Tocilizumab 4 mg/kg + MTX (N=25)	IV Tocilizumab 8 mg/kg + MTX (N=24)	SC Sarilumab 150 mg (N=15)	SC Tocilizumab 162 mg (N=15)
**Neutrophils**						
Baseline, mean±SD, Giga/L	4.32±1.53	4.72±1.96	4.04±1.36	4.52±2.48	3.45±1.11	3.81±0.99
Nadir, mean±SD (min–max), Giga/L	1.55±0.56 (0.61–3.07)	1.76±1.04 (0.21–3.97)	1.78±0.60 (0.96–2.92)	1.64±0.89 (0.69–4.51)	1.11±0.51 (0.3–1.9)	1.78±0.56 (0.9–2.9)
Time to nadir, median, days	3.5	4.0	5.0	3.0	2.0	4.0
**CRP**						
Baseline, median, mg/L	5.50	6.05	4.28	5.87	0.85	0.49
Nadir, median[b] (min–max), mg/L	0.67 (0.10–8.82)	0.49 (0.10–5.13)	0.43 (0.10–1.73)	0.31 (0.10–1.35)	0.06 (0.0–0.2)	0.15 (0.0–0.6)
Time to nadir, median, days	7.0	7.0	15.0	15.0	10.0	10.0
**IL-6**						
Baseline, mean±SD, pg/mL	21.9±58.4	9.86±10.5	7.33±11.1	22.1±45.4	3.97±5.42	5.75±15.5
Peak, mean±SD, (min–max), pg/L	159±356 (22–1844)	177±234 (17–1079)	143±169 (23–644)	183±246 (25–1095)	44.0±42.2 (11–169)	48.4±67.7 (7–231)
Time to peak, median, days	5.0	4.5	6.0	6.5	5.0	7.0
**sIL-6R**						
Baseline, mean±SD, ng/mL	42.9±11.9	42.9±13.7	43.3±14.4	43.8±15.7	36.0±7.9	36.6±11.6
Peak, mean±SD, (min–max), ng/mL	274±72.7 (125–406)	314±72.5 (159–442)	415±109 (230–603)	481±120 (340–704)	265±54.6 (155–344)	291±83.6 (206–469)
Time to peak, median, days	11.0	13.0	19.0	22.0	14.0	14.0
**AEs**						
Patients with TEAEs, N (%)	10 (38.5)	12 (46.2)	8 (32.0)	12 (50.0)	7 (46.7)	4 (26.7)
SAE, N (%)	0	0	0	1 (4.2)	1 (6.7)	0
Patients with ANC <1.0 Giga/L, N (%)	4 (15.4)	7 (26.9)	3 (12.0)	6 (25.0)	5 (33.3)	1 (6.7)
**ANC grade**						
ANC maximum grade: Grade 3 ≥0.5 to <1.0 Giga/L, N (%)	4 (15.4)	6 (23.1)	3 (12.0)	6 (25.0)	2 (13.3)	1 (6.7)
ANC maximum grade: Grade 4 <0.5 Giga/L, N (%)	0	1 (3.8)	0	0	3 (20.0)	0

[a]83.2% of patients were white. [b]Concentrations for CRP were not normally distributed, thus concentrations were summarized by median.

MAIN ADVERSE EFFECTS OF IL-6R BLOCKERS AGENTS AND NEED FOR MONITORING
The most frequently reported adverse reactions with sarilumab treatment in RA patients were: neutropenia, increased liver enzymes (alanine transaminase [ALT]), injection site erythema, upper respiratory tract infections and urinary tract infections.

Adverse reactions of particular interest to consider in this protocol and as exclusion criteria in the candidate population are described below:

- AST / ALT values > 5 times the ULN (ULN),
- Neutropenia: ANC <500 /mm^3
  Neutropenia have not been related to the onset of infections, including serious infections.
- Thrombopenia (less frequent) <50.000/mm^3,
- Infections: special attention to bacterial co-infections
- Diverticulitis: risk of perforation

2.1.2 Experience with IL-6 Receptor Blockers in *SYSTEMIC ACUTE RESPIRATORY SINDROME*, SARS by COVID-19.

In the pathogeny of different SARS produced by different coronaviruses, including COVID-19, high levels of plasma cytokines have been observed, including IL-6, IL-2, IL-7, IL-10, granulocyte-colony stimulating factor (G-CSF), interferon gamma inducible protein (IP10), monocyte chemoattractant protein (MCP1), macrophage inflammatory protein 1 alpha (MIP1A) and TNF-alpha. These findings have been documented in patients found in the Intensive Care Units (ICU) and involve a cytokine storm that is related to the severity of the disease and has prognostic implications. (2-5). The experience of basic and clinical studies in China shows that IL-6 is one of the main engines in this CRS that can lead to dysfunction in the capillary alveolar exchange, especially diffusion of O_2 and eventually to pulmonary fibrosis, organ failure and death. (6). IL-6 blockade has also been proposed as a therapeutic alternative in other CRSs such as sepsis systemic inflammatory reaction syndrome, macrophage activation syndrome, and hemophagocytic lymphohistiocytosis (7).

This evidence, together with the experience of tocilizumab in CAR-T-induced CRS (approved indication), led to the introduction of TCZ iv in clinical treatment protocols for SARS-CoV-2 patients when they had severe lung damage and elevated IL-6 levels (8).

Although the evidence is very scarce, the main data come from the experience in 21 patients treated in China in February 2020 (9), most with severe, non-critical case criteria, defined in China's National Health Commission guidelines (8). 100% of patients had a fever prior to treatment with different degrees of dyspnea. The protocol included the single dose prescription of 400 mg iv of TCZ. 17 patients were classified as severe cases and 4 as critical. 18 received a single dose of TCZ and 3 patients received a new dose of 400 mg iv at 12 hours for fever persistence. Prior to TCZ administration, all patients needed some form of oxygen therapy, but only 1 patient needed non-invasive ventilation and 2 patients invasive mechanical ventilation**.** The results of the study showed a drastic decrease in fever in the first 24 hours and an improvement in $SatO_2$, decreased supplemental oxygen requirements in 75% and extubation of 2 patients between 1 and 5 days. Lymphocyte numbers and CRP improved significantly, as well as procalcitonin. There was an improvement/disappearance of pulmonary infiltrates by 90%, and 90% were discharged after an average hospitalization of 13,5 +/- 3 days. There was no pulmonary overinfection or deterioration of lung disease or deaths. No adverse events related to TZC were reported during the study observation period.

Therefore, this retrospective study shows that, in the vast majority of patients with SARS-Cov2, a single dose of 400 mg iv of TCZ is effective and that its early use in patients with severity criteria, but who do not yet require invasive mechanical ventilation, can modify the evolution of lung disease with very rapid improvement in fever, symptoms, pulmonary infiltrates and oxygenation needs.

The dose recommendations used in this study should be interpreted with caution, as the weight of patients is not described and we cannot rule out that genetic factors or other differential characteristics may be present in the Chinese population.

There are currently at least five clinical trials underway to evaluate the efficacy of different doses of intravenous and subcutaneous TCZ in pneumonia or COVID-19 infection. https://clinicaltrials.gov/ct2/results?cond=COVID&term=tocilizumab&cntry=&state=&city=&dist=

SAR has no health authority approval in CRS for CAR-T, nor does he have clinical experience in the treatment of COVID-19 but clinical trials have already been launched. At least 5 trials

include intravenous and/or subcutaneous SAR in moderate and severe COVID-19 disease, compared to placebo, with antiviral drugs, immunomodulators or intravenous and subcutaneous formulations of TCZ. https://clinicaltrials.gov/ct2/results?term=sarilumab&cond=Covid&draw=1&rank=2#rowId1

2.1.3 EXPERIENCE OF DETERMINING IL-6 LEVELS AS A PROGNOSTIC FACTOR AT THE UNIVERSITY HOSPITAL LA PRINCESA. (Data on File, confidential not share out of Sanofi)

Data on serum IL-6 levels, its association to different clinical and analytical parameters and its prognostic value for outcomes in COVID 19 patients have been submitted and now under review. Therefore, those paragraphs of the rationale have been eliminated.

Considering the phenomenon **"window of opportunity"**(i.e. a period in which the response to treatment is better than when complex perpetuating mechanisms have already been established) the multiple data collected from hospitalized patients in China and the observational study described above, the use of IL-6 inhibitors could be more effective at earlier and reversible stages of disease. These stages would correspond to those defined as moderate and severe early pneumonia in national Health Commission treatment protocols in China (8) or stadium II (moderate) of the therapeutic clinical classification proposed by Siddiqi et al., based on the physiological progression of COVID-19 disease (10). Some authors already propose a time frame for this "therapeutic window" in the use of IL-6R inhibitors, between day 7 and 14 of the onset of symptoms, but the time delimitation, remains to be defined (11).

2.1.4 PROPOSAL FOR USING SUBCUTANEOUS FORMULATION OF SARILUMAB AT UNIVERSITY HOSPITAL LA PRINCESA

General principles:

- Sarilumab is an experimental medicine in the context of the treatment of severe COVID-19 disease and should only be considered in patients with COVID-19 moderate-severe with high risk of CRS, following discussion and multidisciplinary consensus of the specialists involved in the management of these patients.
- SARS and CRS are serious complications of COVID-19 and are associated with high morbidity and mortality.
- Early identification of COVID-19 hyperinflammation using analytical parameters and inflammatory markers (IL-6) is essential for optimizing the use of IL-6R blockers at early stages of pneumonia or moderate-severe infection.
- Every effort should be made to generate experience and evidence with these drugs by collecting results through clinical trials or observational studies with protocolized uses.

Rational for the study at University Hospital La Princesa:

The current epidemiological situation of the COVID-19 pandemic in Madrid, approaching the peak of infected patients, is leading to an exponential increase in inpatient cases that can potentially evolve into SARS (5). The health system is close to saturation, especially critical infrastructure such as emergency and intensive care units (ICUs). High physical and mental pressure on healthcare professionals, with a high number of SARS-CoV-2 infected, decreases the workforce for adequate patient care. On 31st March 2020, the number of patients

admitted, at this centre, by COVID-19 was 577. One of the possible advantages of the proposed intervention is the reduction of hospitalization time and the need for high requirements of oxygen therapy through mechanical ventilation.

2.2 Description and rationale for the route of administration, dose, guideline and treatment period

Given the pharmacokinetic characteristics of sarilumab and tocilizumab, subcutaneous administration of sarilumab may have advantages over tocilizumab sc in COVID-19.
The pharmacodynamic and safety characteristics of a single dose of 150 and 200 mg sarilumab s.c. are known, which do not differ significantly from those obtained with single doses of 4 and 8 mg/kg tocilizumab i.v. (see section 2.A.1). We hope that the administration of two s.c. and earlier-stage injections (moderate or early COVID-19 pneumonia of severe illness), it can increase the initial concentration peak and compensate for the slightly shorter duration of its pharmacodynamic effects of a subcutaneous administration, as described for single dose of 200 mg (1).

We propose a study with randomization sarilumab versus standard of care to include a control group.
As the standard of care evolves very quickly based on clinical learning and available information, protocols are updated with a maximum of 5 days. In our hospital, in the stage of moderate pneumonia [(CURB65≤ 2) and $SatO_2$ >90%] o severe [CURB65>2 or SatO2 <90%] in the early phase, if the patient goes on the inflammatory phase of COVID-19 infection (> 7 days from the onset of symptoms) we can consider starting tocilizumab iv and/or iv methylprednisolone bolus in addition to antiviral treatment and corresponding support.
Tocilizumab will not be accepted in the Standard of Care, previous to randomization in agreement with Exclusion Criteria.

2.3 Study population

Patients with COVID-19 infection treated at University Hospital La Princesa who need hospitalization.

2.4 Declaration that the trial will be conducted in accordance with the protocol, the GCP and the relevant legal requirements

The clinical trial will be conducted with prior authorization from the Spanish Agency for Medicines and Healthcare Products (AEMPS) and the favourable opinion of an Ethics Committees for investigation with medicinal products (CEIm) (see Apendix C). In addition, according to current legislation, a public record of the clinical trial will be conducted prior to the start of patient recruitment.

External monitoring of the trial and data and analysis management will meet the requirements of methodological quality and good clinical practices and other national and international regulations.

This protocol has been considered a clinical trial of low-level intervention because sarilumab is included in the document "Treatments available for the management of SARS-CoV-2 respiratory infection"” of the AEMPS: https://www.aemps.gob.es/la-aemps/ultima-informacion-de-la-aemps-acerca-del-covid%e2%80%9119/tratamientos-disponibles-para-el-manejo-de-la-infeccion-respiratoria-por-sars-cov-2/

Since it is a low-level intervention study, as provided for in AEMPS authorization and current legislation, the clinical trial will be covered by the care civil liability policy of the centre.

3. Study hypothesis

Sarilumab, in subcutaneous formulation, may be effective in treating COVID-19 moderate-severe infection in early stages (window of opportunity) to prevent progression to systemic hyper inflammation status, with serious prognostic implications.

3.1 Study objectives

- To assess the efficacy and safety of early administration of sarilumab subcutaneously in patients with moderate-severe early-stage COVID-19 infection, in comparison to the current standard of care.
- Exploratory: To compare the baseline clinical and biological parameters, including serum IL-6, of the intervention population against historical controls of the same pandemic outbreak (using a propensity score) to search for possible markers that identify candidates for treatment with subcutaneous IL-6 inhibitors (Sarilumab) and to attempt an approximation in the time frame of "window of opportunity"

4. Clinical Study design

4.1 Study Design

Randomized clinical trial, open-label, one site, sarilumab + Standard of Care vs Standard of Care, in a ratio 2:1.

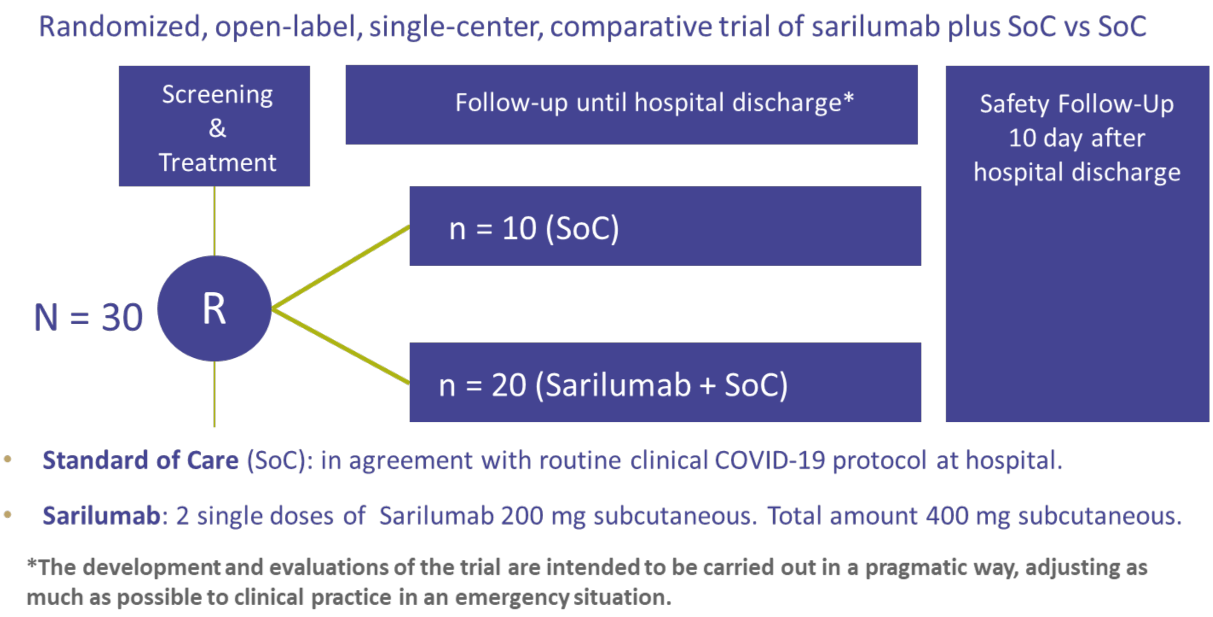

Prospective proof-of-concept study, to evaluate the effect of early administration of sarilumab, in patients with moderate-severe COVID-19 infection compared to a control group receiving the current standard of care.

4.2 Study Endpoints

- **Primary Endpoints**
  - Mean change in the 7-category ordinal scale from study inclusion to **day 7** (after randomization)
    1. Death;
    2. Hospitalized, requiring invasive mechanical ventilation or extracorporeal membrane oxygenation (ECMO);
    3. Hospitalized, requiring non-invasive ventilation or high flow oxygen devices;
    4. Hospitalized, requiring supplemental oxygen;
    5. Hospitalized, not requiring supplemental oxygen - requiring ongoing medical care (COVID-19 related or otherwise)
    6. Hospitalized, not requiring supplemental oxygen - no longer requires ongoing medical care (independent)
    7. Non-hospitalized

  - Duration of hospitalization (days)
  - Mortality rate at the end of the study
- **Secondary Endpoints**
  - Time to become afebrile for a minimum period of 48 hours, without antipyretics.
  - Time to non-invasive mechanical ventilation (days)
  - Time to invasive mechanical ventilation (days)
  - Time to withdraw oxygen therapy (days)
  - Mean change in the 7-category ordinal scale from study inclusion to **day 14** (after randomization)

- **Safety endpoints**
  - Incidence of serious and non-serious adverse reactions.
  - Incidence of serious and non-serious adverse events.
  - Discontinuation due to adverse reactions

4.3 Randomization process

Randomization to treatment with sarilumab or standard treatment in a 2:1 ratio, will be performed by the Clinical Research and Clinical Trials Unit (CRCTU) at the Hospital using a table of random numbers. Each time the researchers include a patient they will call to the CRCTU, who after checking that all inclusion criteria are met and none of the exclusion criteria, will tell them the treatment assigned.

4.4 Masking process

Non-applicable

4.5 Treatment period

Single dose

4.6 Schedule of clinical trial evaluations

STRICTLY CONFIDENTIAL: Property of Hospital Universitario de la Princesa.
It is not allowed the dissemination or replication out of terms of this proposal

The expected duration of subject participation in the trial is up to the resolution of COVID-19 infection, approximately 1 month. The overall duration of the study is estimated at about 2 months, from the recruitment of the first patient (FPV) to the completion of the follow-up of the last patient included (LPLV). The duration may be shortened if the current income rate of hospitalized patients is maintained for at least one month.

4.7 Data Management

The data obtained will be entered by the researchers in an electronic data collection sheet (eCRF). The identification of the subjects included in the clinical trial shall be made by a code composed of the trial code followed by the patient's number, separated by a hyphen (SARCOVID-XX). The responsible physician, in turn researcher, will be the only one who has access to the relationship of the code with the patient's medical history.

Definition of end of Study

The end of the trial is defined as the last visit of the last patient (LPLV).

5. Study population

30 patients attended at the University Hospital La Princesa, by COVID-19 infection requiring hospitalization will be recruited, meeting all inclusion criteria and none of the exclusion criteria.

5.1 Inclusion criteria

a. Age >18 , <80 years old
b. COVID-19 infection documented by a positive RT-PCR test or, in absence of a RT-PCR positive test, case definition of COVID 19 infection/pneumonia as per local protocol and the presence of a positive serologic test (IgM/IgA assessed by ELISA)
c. Documented interstitial pneumonia requiring admission and at least two of the following parameters:
    1) Fever ≥ 37.8ºC (tympanic)
    2) IL-6 in serum ≥ 25 pg / mL (in the absence of a previous dose of prednisone or equivalent> 1 mg / kg) or PCR> 5mg / dL
    3) Lymphocytes <600 mm^3
    4) Ferritin> 300 μg /L twice in 24 hours
    5) Ferritin> 600 μg /L in the first determination and LDH> 250 U/L
    6) D-dimer (> 1 mg / L)
d. Informed verbal or administration consent under urgent conditions, documented in the medical record.

5.2 Exclusion criteria

a. Patients who require mechanical ventilation at the time of inclusion.
b. AST / ALT values > 5 times the ULN.
c. Absolute neutrophil count below 500 cells / mm^3
d. Absolute platelet count below 50,000 cells / mm^3
e. Documented sepsis or high suspicion of superimposed infection by pathogens other than COVID-19.
f. Presence of comorbidities related, according to clinical judgment, with an unfavorable result.

STRICTLY CONFIDENTIAL: Property of Hospital Universitario de la Princesa.
It is not allowed the dissemination or replication out of terms of this proposal

g. Complicated diverticulitis or intestinal perforation.
h. Current skin infection (eg, uncontrolled dermopiodermitis).
i. Immunosuppressive anti-rejection therapy.
j. Pregnancy or lactation.
k. Previous treatment with tocilizumab or sarilumab.
l. Patients participating in some other clinical trial for SARS-CoV-2 infection.
m. Patients with known hypersensitivity or contraindication to sarilumab or excipients.

5.3 Withdrawal and analysis criteria

Patients may be withdrawal from the study for any of the following reasons:

a) By patient's own decision at any time during the study.
b) For any adverse event or circumstance that at the discretion of the researcher may compromise the health and well-being of the patient.
c) In cases where the patient does not cooperate or does not meet the requirements of the study.
d) At the discretion of the investigator.

In either case, the information obtained about the patient must be recorded until the time of withdrawal.

Eligible patients (who meet the inclusion criteria) will be included in the "intent to treat" analysis whether they strictly follow the protocol. In the analysis "per protocol" only those patients who have successfully completed treatment and follow-up will be assessed.

Patients who have signed the informed consent and who for different reasons (withdrawal of consent, decision of the researcher, etc.) have not received the dose of medication in the study, will be substituted to reach the calculated sample size. These patients will be considered as loss not valued for statistical analysis.

6. Patients treatment

6.1 Definition of treatment

Kevzara 200 mg SC, 2 injections in pre-filled syringe, single dose (400 mg).

6.2 Procedure to monitor subject compliance

Not apply, as the treatment will be administered at the study center by the nurse in charge of the patient.

6.3 Interaction with other medicines and other forms of interaction

Drugs or procedures in the usual clinical practice according to the local protocol are permitted when the physician responsible for the patient deems necessary.

7. Study development

7.1 Study visits

The development and evaluations of the trial are intended to be carried out in a pragmatic manner, as much as possible to clinical practice in an emergency situation such as the current. All evaluations are considered routine and whenever possible they will be obtained from the Electronic Clinical History.

The study procedures do not involve any examination or procedure in addition to the usual clinical practice in the care of patients with COVID-19 at hospital level. All patients will be determined for serum IL-6 levels at admission and 5-7 days after the dose. Occasionally it will be repeated at 10-14 days after first dose. In all patients, quantiferon and HBV and HIV serologies are previously performed, although it is not necessary to wait for the result to administer the study medication.

The selection visit and treatment visit will take place on the same day. Patients will be evaluated daily during the check-in period and a phone call will be made 10-15 days after discharge to check the patient's situation.

7.2 Study Calendar and Procedures

**Procedure**	**Selection visit***	**Treatment visit***	**Follow-up until hospital discharge**	**Final visit 10-15 Day after discharge**
Selection criteria	X			
Informed consent	X			
Physical examination and vital signs	X	X	According to clinical practice	
Documentation of respiratory status	X	X	X	X
Radiological findings	X		According to clinical practice	
Recording PCR results for SARS-COV-2	X		According to clinical practice	
Blood tests (blood count, biochemistry and coagulation)	X		According to clinical practice	
Quantiferon	X**			
Serology HIV, VHB, (viral load if positive)	X**			
IL-6	X		Day 5 after treatment and if patient is still hospitalized at 10-14 day	
Treatment with sarilumab sc		X		
Adverse event log		X	X	X
Concomitant medication record	X	X	X	X
Safety visit by Phone				X

* Usually the selection and treatment visit will be made on the same day
** No need to wait for the result to administer treatment

7.3 Quality Control - Monitoring

During the recruitment period, the clinical trial monitor will check the degree of cooperation and qualification of the researchers and staff involved in conducting the study and selecting patients.

In the course of the study, the monitor is responsible for the smooth running of the study and for carrying out quality controls of the study. It will examine all documentation, determining its validity and monitoring of the protocol. The occurrence of side effects will also be assessed.

7.4 Collecting clinical data

The patient's full physical examination, as well as the collection of information from their medical history, will be performed at the basal visit. Data on sociosanitary situation will also be collected and a functional assessment will be carried out. The following sections record the parameters described in the study as well as any variations in the patient's situation.

8. Efficacy measures

For the evaluation of the response to treatment, the following events will be collected:

- **Primary Endpoints**
  - Mean change in the 7-category ordinal scale from study inclusion to **day 7** (after randomization)
    1. Death;
    2. Hospitalized, requiring invasive mechanical ventilation or extracorporeal membrane oxygenation (ECMO);
    3. Hospitalized, requiring non-invasive ventilation or high flow oxygen devices;
    4. Hospitalized, requiring supplemental oxygen;
    5. Hospitalized, not requiring supplemental oxygen - requiring ongoing medical care (COVID-19 related or otherwise)
    6. Hospitalized, not requiring supplemental oxygen - no longer requires ongoing medical care (independent)
    7. Non-hospitalized
  - Duration of hospitalization (days)
  - Mortality rate at the end of the study
- **Secundary Endpoints**
  - Time to become afebrile for a minimum period of 48 hours, without antipyretics.
  - Time to non-invasive mechanical ventilation (days)
  - Time to invasive mechanical ventilation (days)
  - Time to withdraw oxygen therapy (days)
  - Mean change in The 7-category ordinal scale from study inclusion to **day 14** (after randomization)
- **Safety end points**
  - Incidence of serious and non-serious adverse reactions.
  - Incidence of serious and non-serious adverse events.
  - Discontinuation due to adverse reactions

9. Safety measures.

Safety assessments will be conducted in accordance with the Standards of Good Clinical Practice and current legislation. The sponsor is responsible for handling adverse events (AE) collected during the study. Expedited notification to Health Authorities (AEMPs, Autonomous regions) and the ethics committee (CEIm) of serious and unexpected adverse reactions (SUAR), annual reports or any other relevant security information, will be the responsibility of the sponsor. Likewise, the sponsor is responsible for the notification of safety information to investigators.

9.1 Definitions

Adverse event (AE): It is any harmful incidence of health in a patient or subject of a clinical trial treated with a drug, even if it does not necessarily have a causal relationship with such treatment. An AE may therefore be any unfavorable and unintended signs (including an abnormal laboratory finding), symptom or illness temporarily associated with the use of a subject drug, whether or not it is related to the investigation drug.

Adverse reaction (AR): Any harmful and unintentional reaction to an investigation drug, regardless of the dose administered, is considered an adverse reaction. Unlike an AE, in the case of an adverse reaction there is a suspected causal link between the investigation drug and the adverse event. All AE and AR will be classified based on their intensity as mild, moderate or intense.

Intensity Criteria: The intensity will be defined as specified below:
- Mild: patient experiences discomfort that doesn't interfere with everyday activity.
- Moderate: patient experiences enough discomfort to reduce or affect everyday activity.
- Severe: patient suffers inability to work or carry out everyday activity.

The determination of the possible relationship with the treatment of the study is the responsibility of the principal investigator of the research center or the person designated by the research center.

Imputability criteria: The sponsor will classify adverse events, based on their causal relationship with the drug, in accordance with the Algorithm of the Spanish Pharmacovigilance System, as:
- Definitive: there is a reasonable time sequence between the administration of the drug and the onset of the adverse event. This event coincides with the adverse reactions described for the drug, improves with the suppression of the drug, reappears after re-administration and cannot be explained by alternative causes.
- Probable: there is a reasonable time sequence between the administration of the drug and the onset of the adverse event. This event coincides with the adverse reactions described for the drug, improvement after discontinuation of treatment and cannot be explained by other alternatives.
- Possible: there is a reasonable time sequence between the administration of the drug and the onset of the adverse event. This event coincides with the adverse reactions described for the drug, but can be explained by alternative causes.

STRICTLY CONFIDENTIAL: Property of Hospital Universitario de la Princesa.
It is not allowed the dissemination or replication out of terms of this proposal

- Conditional: there is a reasonable time sequence between the administration of the drug and the onset of the adverse event. This event does not match the adverse reactions described for the drug and can be explained by alternative causes.
- Unrelated: there is no reasonable time sequence between the administration of the drug and the onset of the adverse event. This event does not match the adverse reactions described for the drug and can be explained by alternative causes.

For expedited notification purposes, the categories shall be considered to be related: definitive, probable and possible, and as unrelated to the conditional category of that algorithm.

Serious Adverse Event (SAE) y Serious Adverse Reaction (SAR): Any adverse event or adverse reaction that, at any dose:
- cause the patient to die.
- threatens the patient's life.
- require hospitalization or extension of the patient's hospitalization.
- cause permanent or significant disability or disability.
- result in an anomaly or congenital malformation.

For the purposes of notification, suspected adverse events or adverse reactions deemed to be medically important, even if they do not meet the above criteria, including important medical events requiring intervention to prevent any of the consequences described above, shall also be treated as serious. Likewise, all suspicions of transmission of an infectious agent through an infectious medicinal product shall be reported as serious.

The concept of "severe (serious)" should not be confused, described above, with “Intense (severe)” which refers to the intensity of the AE or AR (mild/moderate/intense).

Serious and Unexpected Adverse Reaction (SUAR): Any serious adverse reaction whose nature, intensity or consequences do not correspond to the reference information for the medicinal product (Kevzara SmPC®). The unexpected nature of an adverse reaction is based on the fact that it has not been previously observed and will not be based on what might be anticipated based on the pharmacological properties of the medicinal product.

9.2 Security Reference Information

The Safety Reference information for the clinical trial corresponds to the section **"4.8 Adverse Reactions"** of the Kevzara SmPC®, published on the website of the Spanish Medicines and Healthcare Products Agency (AEMPS).

9.3 Collecting adverse events/pregnancies

AE/pregnancy will be collected since the patient signs the Informed Consent up to 30 days after administration of the investigation drug dose. In case of pregnancy, the patient will be excluded from the study. All AE/pregnancy must be documented in the patient's medical history and case report form (CRF).

All reported adverse events/pregnancies, either spontaneously by the patient or during interviews with the patient at the study visits, will be collected. Information concerning AEs

STRICTLY CONFIDENTIAL: Property of Hospital Universitario de la Princesa.
It is not allowed the dissemination or replication out of terms of this proposal

that occurred in the period of time between the previous visit and the current visit should be requested.

All AE should be tracked until resolution, or at least 30 days after discontinuation of the drugs in the study, (depending on what occurs first).
Any pregnancy that occurred during the study and its outcome should be recorded and followed to rule out birth defects or malformations. Information on:

- Normal birth, miscarriage or therapeutic (any congenital abnormalities detected in the aborted fetus should be documented), stillborn fetus or congenital abnormality.
- Neonatal deaths occurring in 30 days from birth.
- Death of an infant after 30 days if the investigator suspects that it is related to intrauterine exposure to the study medication.
- All infants born after fetal exposure should be followed for the first 12 months after delivery.

Any exacerbation of a pre-existing condition that occurs after the start of study treatment is also considered an adverse event.

Any abnormal results in analytical tests that the researcher considers clinically significant and that requires the transient or permanent discontinuation of the investigational medicinal product, any type of intervention or diagnostic evaluation to assess the associated risk to the patient, will be collected as an adverse event, to be properly investigated and monitored.

9.4 SAE that do not require notification to the Pharmacovigilance Unit

It is not necessary to notify the Sponsor's Pharmacovigilance Unit immediately of the following SAE:

- Hospitalization or death due to disease progression.
- Hospitalization for scheduled testing.
- Hospitalization to provide palliative care, terminal care or for scheduled surgical interventions.
- Admission to the hospital emergency department (<24 h) for clinical observation.

Nonetheless, they will be collected in the CRF and in the patient's medical history. The rest of SAEs and all pregnancy cases should be reported according to the procedure described in the following item.

9.5 Procedure for notification of SAE/pregnancies to the Promoter

In the event of an SAE to be notified or a pregnancy case is collected, a member of the investigating team will complete and sign the SAE or pregnancy notification form, which will be faxed immediately and always within 24 hours of becoming aware of the event:

Unidad de Farmacovigilancia
UICEC Hospital La Princesa
Fax: 91 520 24 25

The form received will be reviewed and, if applicable, additional information will be requested from the investigator. Where additional information on the SAE/pregnancy is obtained, or

resolved or unlikely to be changed, a follow-up report shall be completed and also faxed to the Pharmacovigilance Unit.

If SAE is suspected of being a SUAR, the investigator should provide the follow-up information requested by the Pharmacovigilance Unit. Any SAE that occurs more than 30 days after the end of treatment (no time limit) should be notified if the researcher considers that the SAE is related to the treatment of the study (i.e. if it is a serious adverse reaction) or if it is medically important.

9.6 Expedited notification of SUAR to Health Authorities/CEIm

The Pharmacovigilance Unit of the Sponsor is responsible for notifying AEMPS (Area of Clinical Trials of the General Deputy Direction of Medicinal Products for Human Use), CEIm and the Autonomous Community of Madrid, all SUARs collected in the study, following the procedure indicated in the current legislation.

9.7 Notification deadlines

The maximum period of notification of an individual case of SAE suspicion shall be 15 calendar days from the time when the sponsor became aware of it. Where the suspicion of SAE has caused the death of the patient, or endangered his life, the sponsor will send the information within 7 calendar days from the time he becomes aware of it. You will complete this information, where possible, within 8 days.

9.8 Expedited notification of other relevant security information

The sponsor shall also provide, expeditiously, with any information that could modify the benefit/risk balance of the investigational medicinal product, or determine changes in its administration schedule or in the conduct of the trial, such as:

- A qualitative change or an increase in the percentage of occurrence of expected SAEs, which is considered clinically important.
- SAEs that occur after the completion of the study and are notified by the researcher to the sponsor.
- New developments related to the conduct of the trial or the development and likely to affect the safety of patients such as:
    - SAE that may be associated with the testing procedures and may modify the performance of the test.
    - A significant risk to subjects such as ineffectiveness in a drug used to treat a life-threatening disease.
    - Important new safety findings from new animal studies (such as carcinogenicity).
    - Any premature completion or temporary stop of a clinical trial with the same drug under investigation for safety reasons, performed in another country by the same sponsor.

This relevant information will be notified as soon as possible and no later than 15 days after the sponsor has become aware of it. Additional information will also be reported as quickly as possible.

9.9 Annual safety report

The annual safety report that will include SUARs and SAEs collected in the study, will be sent by the Sponsor to the AEMPS (Clinical Trials Area of the general deputy direction of Medicinal

STRICTLY CONFIDENTIAL: Property of Hospital Universitario de la Princesa.
It is not allowed the dissemination or replication out of terms of this proposal

Products for Human Use), the CCAA and CEIm, within the deadlines set out in the current legislation.

9.10 Notification to investigators

The Sponsor shall communicate to the investigators any safety information that may affect the safety of the trial subjects, as soon as possible. Information on SUARs will be sent annually, in aggregate, in a list together with a brief analysis of the data provided. In the case of blind studies, the listings will present SUARs globally and not broken down by medication, so as not to reveal treatment to researchers. You will also be informed, throughout the study, of any safety aspects that influence the completion of the clinical trial or the development of the product, including disruption of the development program or modifications to the protocol related to safety.

9.11 General and particular standards for researchers

If during the study any patient suffers from a disease or AE that in itself or by requiring pharmacological treatment could modify the disposition of the study drug, or make its administration inadvisable, the patient will be excluded from the study, detailing the cause. If the AR was mild or moderate, whether or not it required treatment, and did not involve discontinuation of the drug, this reaction would be reported in detail and the study would continue as planned.

10. Statistical analysis

10.1 Data management

The data obtained will then be entered by researchers or collaborators in a database, dissociated from clinical information by means of a code (ID). The trial code, followed by the patient's number, separated by a hyphen, will be used for coding (SARCOVID-XX). The responsible physician, in turn researcher, will be the only one who has access to the ID relationship with the patient's medical history. The data entered in the database will be encrypted and the database will be password protected, to which only researchers will have access.

A data validation plan will be developed to verify and control existing inconsistencies with respect to the action plan described in the protocol, as well as to detect possible lost data. These filters will be applied after the data is entered into the database. Before carrying out the statistical analysis, the database will be refined and validate the data of the database as a control process, with special emphasis on the main objectives of the study, to ensure the quality of the data and the results. Once the database is cleaned, it will be closed and frozen to proceed with the analysis of the data.

10.2 Description of statistical methods

The number of patients who have violations of the protocol and/or do not meet the inclusion criteria will be indicated, breaking down the reasons, thus obtaining the final sample valid for the analysis.

STRICTLY CONFIDENTIAL: Property of Hospital Universitario de la Princesa.
It is not allowed the dissemination or replication out of terms of this proposal

A general description of the characteristics of the patients and the injections (dose, location of administration, ...) will be made. The categorical variables (sex, race, background, etc.) will be described by means of their distribution of absolute and relative frequencies, while the continuous variables (age,…) will be described by means of measures of central tendency and dispersion, that is: mean, median , standard deviation, minimum and maximum.

The statistical analysis will be performed using the STATA v14 statistical package. It will be confirmed by means of standard statistics of parametric and non-parametric distributions that there are no significant differences in demographic, clinical and analytical variables at admission between the intervention population and the control population.
For the study of prognostic biomarkers, a comparison will be made with a historical cohort, from the same pandemic in the same hospital, selecting 3 controls paired by propensity score for each intervention patient.
Given the large number of confounding variables that exist in the populations under study, multivariable analysis techniques based on generalized linear models will be used (STATA glm command) that allow a looser approximation than multivariable linear regression models. The main variable, death, will be analyzed using multivariate logistic regression with the family (binomial) and link (log) options of glm. The other main variables (mean change in clinical status using the 7-point ordinal scale and length of hospitalization) will be analyzed using multivariate linear regression with the glm family (gaussian) and link (identity) options. In the initial models, all the variables considered clinically relevant (sex, age, viral load on admission, time until administration of the IL6R blocking treatment, IL6 levels, CRP, etc.) that reach a p value <0.1 in the bivariate analysis will be included.
For the analysis of the secondary variables (Time to become afebrile, Time to non-invasive mechanical ventilation, Time to invasive mechanical ventilation, Time to withdrawal of oxygen therapy) the use of incidence models such as the Poisson regression or its variant will be evaluated. " Zero-inflated Poisson regression” depending on the behavior of the variables.

10.3 Sample Size

As this is a proof of concept study with a drug that has not been previously evaluated in this pathology, a formal calculation of the sample size has not been performed, but it is thought that 30 patients (20 sarilumab: 10 control) may be sufficient to make an initial evaluation of the study objectives. If the results are favorable, then a phase III study with a larger sample size calculated based on the expected benefits with this treatment should be done.

For ethical reasons, it does not seem reasonable to include a placebo-controlled arm, so controls will receive standard of care for comparison will be used.

11. Direct access to data/source documents

The sponsor shall ensure that it is specified in the protocol or other written agreement that the investigator or the institution will allow direct access to the source data or documents for monitoring, auditing, review by the CEIm, as well as the inspection of the trial by the health authorities.

The information disseminated and obtained by the implementation of this study is considered confidential and should be treated at all times as such. Study subjects will be identified with a subject number. Both the researchers responsible for the clinical trial, a representative of the

sponsor or the Health Authorities will have access to the information recorded throughout the study. In case of publication of the results of the study, the identity of the patients will not be disclosed.

All information collected in the data collection file (CRF) must be present at the primary source (electronic medical history). Any changes to the medical history should be traceable and attributable.

12. Quality control and assurance

During the course of the study, the sponsor or his representatives will make regular monitoring visits to ensure that the protocol and standards of Good Clinical Practice are followed. Monitors will be able to review the original documents to confirm that the data recorded in the CRDs are accurate. The investigator and the centre shall allow the Sponsor's monitors or their representatives and the competent health authorities direct access to the original documents for such verification. The centre of the study may be reviewed by CEIm or quality assurance audits carried out by the Sponsor or by undertakings working with or on behalf of the Sponsor, or inspections by the competent health authorities. It is important that researchers and their relevant staff are available during monitoring visits and possible audits or inspections and that they spend sufficient time on the process.

12.1 Deviations from protocol

When a situation occurs that causes a deviation from the protocol, the deviation will be for that patient only. Investigators present in such circumstances shall fully document the deviation and reason in the data collection notebook. In the event that the deviation relates to the inclusion/exclusion criteria, the researchers will contact the clinical monitor by telephone to inform you of such deviation.

13. Ethics

13.1 General and particular standards for researchers

The researchers will strictly abide by the provisions of this protocol, fully completing the data collection sheets, which will be sent in due time to the sponsor or the collaborating entity that it designates to analyze the data. The trial will be conducted in accordance with the recommendations for clinical trials and drug evaluation in humans, as contained in the Helsinki Declaration (Appendix A),revised in Tokyo, Venice, Hong Kong, South Africa, Edinburgh, Washington, Tokyo, Seoul and Strength (2013) and the current Spanish Legislation on Clinical Trials.

Auxiliary staff will follow the instructions given by the researcher regarding blood sample extractions, handling and other additional scans. Prior to the study, patients should receive oral and written information regarding the design, purposes of the study and possible risks that may arise from it. If they subsequently agree to participate in it, they must sign their consent, without preventing them at any time and for any reason from being able to revoke it and leave the study. Patients will be instructed on the need to strictly follow the instructions of the researchers. Patients will be informed of the need to contact the researchers if any incidents arise during the study, providing them with how to do so during the outpatient period of the study. If during the study any patient suffers any adverse disease or event that in

itself or by requiring drug treatment could modify the disposition of any of the drugs under study, or makes it ill-advised to administer, the patient will be excluded from the study by consigning in detail the cause. If the adverse reaction was mild or moderate, requires or not to be treated, and did not involve discontinuation of the drug, such a reaction would be recorded in detail and the study would continue as planned.

13.2 Ethics Committees for investigation with medicinal products (CEIm)

The investigator shall be responsible for obtaining prior approval of the study protocol from CEIm, amendments to the protocol, informed consent documents and other relevant documents if applicable. All correspondence with CEIm must be stored in the investigator's file. Copies of CEIm approvals shall be forwarded to the sponsor. An amendment may only be initiated without the prior approval of CEIm where the change is necessary to eliminate obvious immediate risks to patients. In such a case, the investigator shall notify CEIm and sponsor immediately in writing after its application.

13.3 Informed consent

The CI document shall be in accordance with the ICH PCB, local administrative rules and legal requirements. The informed consent document used in this study and any changes made to it during the study must be approved in advance by CEIm and the sponsor prior to use.

The researcher should ensure that all patients in the study, or their legal representatives, are fully informed of the nature and objectives of the study and of the potential risks associated with participation. The investigator or the person he/she designates will obtain the informed written consent of each patient or his/her legal representative before performing any specific activity of the study and a copy of it will be given to the patient or his/her legal representative to keep it. The investigator will keep the original of all consent documents signed by patients.

13.4 Amendments to the protocol

Neither the principal investigator nor the sponsor will modify this protocol without first obtaining the consent of the other parties. The modification must be documented in writing. Any changes in research activity, except those necessary to eliminate an immediate apparent risk to the patient, must be reviewed and approved by CEIm prior to implantation. The sponsor must send the amendments to the protocol to the health authorities, and the amendments may require the revision and approval of the CEIm.

13.5 Security and confidentiality devices

The information disseminated and obtained by the implementation of this study is considered confidential and should be treated at all times as such. The subjects of the study will be identified by a subject number. Both the researchers responsible for the clinical trial, a representative of the sponsor or the Health Authorities will have access to the information recorded throughout the study. In case of publication of the results of the study, the identity of the patients will not be disclosed.

14. Handling data and archiving records

14.1 Documentation Archive

There will be a documentation file for all data, which will be kept in full on computer support for 25 years after the completion of the study. This file should contain the following elements:

1. CEIm's approval of the protocol and informed consent sheet.

STRICTLY CONFIDENTIAL: Property of Hospital Universitario de la Princesa.
It is not allowed the dissemination or replication out of terms of this proposal

2. Copy of the written consent form, and protocol approved with any amendment if appropriate.
3. Any correspondence relating to the study with the sponsor, during the course of the same.
4. Any correspondence with CEIm.
5. Signed acceptance of the protocol.
6. Curriculum vitae of the principal investigator and the other researchers who form the research team.
7. Record signatures of research team members.
8. Delegation of the tasks of the research team.
9. Communications of serious adverse events.
10. Contract between the sponsor and the research team.
11. Copies of data collection notebooks.

Documentation will be archived according to the Standards of Good Clinical Practice.

14.2 Case report form (CRF) / data record

The CRF will be filled in by data managers and all study variables will be collected from the clinical information recorded and hosted in different applications of the electronic medical history (HCIS).

For the purposes of this protocol, CRF means an electronic data record or both, depending on the method of data collection used in this study. A CRF should be filled in for each patient included in the study. The data included in the CRF are the exclusive property of the Sponsor and should not be available in any way to third parties, except the authorized representatives of the Sponsor or the competent health authorities, without the written authorization of the Sponsor.

The investigator shall have the ultimate responsibility for collecting and notifying all clinical, security and analytical data entered into the CRF and any other data collection document (original documents), and for ensuring that they are accurate, authentic/original, attributable, complete, consistent, legible, current and durable and available when needed. The CRF will be signed by the researcher or an authorized team member to attest that the data it contains is true. All corrections made to the CRF annotations or original documents must be dated, signed with the initials and explained (if necessary), and may not hide the original annotation. In most cases, the original documents are the medical records of hospital or physician patients. In these cases, the data included in the CRFs must match those in those stories.

In some cases, the CRF or part of it may also serve as an original document. In such cases, a document clearly identifying the data to be recorded in the CRF and for which the CRF will serve as an original document must be available at both the investigator's centre and the Sponsor.

14.3 Medication identification

The medication of the study will be provided by Sanofi and labeled in accordance with the recommendations of Royal Decree 1090/2015.

15. Insurance

Because it is a low-level study of intervention and in accordance with current legislation (RD 1090/2015 of December 4) the clinical trial will be covered by the center's assistance liability policy, provided that AEMPS so dictates.

16. Publishing policy

The principal investigator and coordinator are committed to publishing the results, both positive and negative, in scientific journals. Researchers and collaborating staff are required not to transmit to third parties, disclose or publish the results of the project without the written consent of Dr. García de Vicuña and Dr. Sanz Sanz
In any case, the following conditions must be observed:

- Project results cannot be published until completion, unless relevant findings prior to completion advise it.
- The sponsor will not cite the names of the investigators without their consent, except in the case of reference to works already published.
- Dr. García de Vicuña, and Dr Sanz Sanz allow the publication of the data obtained in the project in scientifically prestigious journals and their dissemination in seminars and conferences within the medical professional field, provided that the above established is respected.
- If, after completion of the project, one or more researchers agree to perform other analyses with the data collected from the patients, they may only do so with the authorization of the sponsor, and the project coordinator researcher, accepting the points specified above and provided that they cite all researchers participating in possible publications.
- The criteria for authoring publications will be those detailed in the ICMJE (http://www.icmje.org/) ("Uniform Requirements for Submission of Manuscripts to Biomedical Journals")
- All researchers designated as "authors" must meet the qualification of author. Each author must have participated in the work in a significant way so that he can take public responsibility for his or her authorship. A substantial contribution to the project is supported by:
- Development in the concept and design, analysis or interpretation of data.
- Writing the draft article or critical review of it in terms of the intellectual relevance of its content.
- Approval of the final version of the article to be published.
- The above three paragraphs must be complied with. All researchers will be included in the research group in all publications generated from this project. The coordinating researchers and the sponsor will also be listed as authors.
- The researchers of the project will be signatories of the scientific articles and communications to congresses derived from this project, as authors. The order of authorship will be established based on the number of patients recruited.

STRICTLY CONFIDENTIAL: Property of Hospital Universitario de la Princesa.
It is not allowed the dissemination or replication out of terms of this proposal

References

1. Ishii T, Sato Y, Munakata Y, et al AB0472 Pharmacodynamic effect and safety of single-dose sarilumab sc or tocilizumab iv or sc in patients with rheumatoid arthritis (RA) Annals of the Rheumatic Diseases 2018;77:1397-1398
2. Chen N, Zhou M, Dong X, et al. Epidemiological and clinical characteristics of 99 cases of 2019 novel coronavirus pneumonia in Wuhan, China: a descriptive study. Lancet 2020; 395(10223):507-513.
3. Wang D, Hu B, Hu C, et al. Clinical Characteristics of 138 Hospitalized Patients With 2019 Novel Coronavirus-Infected Pneumonia in Wuhan, China. JAMA 2020. DOI: 10.1001/jama.2020.1585
4. Huang C, Wang Y, Li X, et al. Clinical features of patients infected with 2019 novel coronavirus in Wuhan, China. Lancet 2020;395:497-506
5. Zhou F, Yu T, Du R et al. Clinical course and risk factors for mortality of adult inpatients with COVID-19 in Wuhan, China: a retrospective cohort study. Lancet 2020; 395: 1054–62
6. Gao Y, Li T, Han M, Li X, Wu D, Xu Y, Zhu Y, Liu Y, Wang X, Wang L. Diagnostic Utility of Clinical Laboratory Data Determinations for Patients with the Severe COVID-19. J Med Virol. 2020 PMID: 32181911.
7. Bracaglia C, Prencipe G, De Benedetti F. Macrophage Activation Syndrome: different mechanisms leading to a one clinical syndrome. Pediatr Rheumatol Online J. 2017 Jan 17;15(1):5.
8. National Health Committee of the People's Republic of China. China's National Health Commission treatment guidelines 7th version [Internet]. Beijing; 2020. https://www.chinalawtranslate.com/wp-content/uploads/2020/03/Who-translation
9. Xu X, Han M, Li T, Sun W, Wang D, Fu B, et al. Effective Treatment of Severe COVID-19 Patients with Tocilizumab. ChinaXiv [Internet]. 2020 Mar 5 [cited 2020 Mar 16]; Available from: https://www.ser.es/wp-content/uploads/2020/03/TCZ-and-COVID-19.pdf
10. Siddiqi, HK et al. COVID-19 Illness in Native and Immunosuppressed States: A Clinical-Therapeutic Staging Proposal. J Heart Lung Transp https://doi.org/10.1016/j.healun.2020.03.012
11. de Ferro F, Elefante F, Baldini C et al COVID-19 : the new challenge for rheumatologists.Clin Exp Rheumatol. 2020 Mar-Apr;38(2):175-180. Epub 2020 Mar 24.
12. Chaomin W, Risk Factors Associated With Acute Respiratory Distress Syndrome and Death in Patients With Coronavirus Disease 2019 Pneumonia in Wuhan, China. JAMA Intern Med. Published online March 13, 2020. doi:10.1001/jamainternmed.2020.0994
13. Clinical management of severe acute respiratory infection when Novel coronavirus (2019-nCoV) infection is suspected: Interim Guidance https://www.who.int/docs/default-source/coronaviruse/clinical-management-of-novel-cov.pdf
14. Handbook for the care of people with COVID-19 disease (Italy) http://www.simit.org/medias/1568-covid19-vademecum-20-13-marzo-2020.pdf
15. The University of Michigan Inpatient Guidance for diagnosis & treatment of COVOD-19 in adults & children http://www.med.umich.edu/asp/pdf/adult_guidelines/COVID-19-treatment.pdf
16. https://www.ser.es/wp-content/uploads/2020/03/nota_Distribucion-medicamentos-antivirales_COVID-19_16032020.pdf

APPENDIX A: Helsinki declaration WMA

Ethical principles for medical research involving human subjects

Adopted by the 18th WMA General Assembly, Helsinki, Finland, June 1964
and amended by the:
29th WMA General Assembly, Tokyo, Japan, October 1975
35th WMA General Assembly, Venice, Italy, October 1983
41st WMA General Assembly, Hong Kong, September 1989
48th WMA General Assembly, Somerset West, Republic of South Africa, October 1996
52nd WMA General Assembly, Edinburgh, Scotland, October 2000
53rd WMA General Assembly, Washington DC, USA, October 2002 (Note of Clarification added)
55th WMA General Assembly, Tokyo, Japan, October 2004 (Note of Clarification added)
59th WMA General Assembly, Seoul, Republic of Korea, October 2008
64th WMA General Assembly, Fortaleza, Brazil, October 2013

Preamble

1. The World Medical Association (WMA) has developed the Declaration of Helsinki as a statement of ethical principles for medical research involving human subjects, including research on identifiable human material and data.

The Declaration is intended to be read as a whole and each of its constituent paragraphs should be applied with consideration of all other relevant paragraphs.

2. Consistent with the mandate of the WMA, the Declaration is addressed primarily to physicians. The WMA encourages others who are involved in medical research involving human subjects to adopt these principles.

General Principles

3. The Declaration of Geneva of the WMA binds the physician with the words, “The health of my patient will be my first consideration,” and the International Code of Medical Ethics declares that, “A physician shall act in the patient’s best interest when providing medical care.”

4. It is the duty of the physician to promote and safeguard the health, well-being and rights of patients, including those who are involved in medical research. The physician’s knowledge and conscience are dedicated to the fulfilment of this duty.

5. Medical progress is based on research that ultimately must include studies involving human subjects.

6. The primary purpose of medical research involving human subjects is to understand the causes, development and effects of diseases and improve preventive, diagnostic and therapeutic interventions (methods, procedures and treatments). Even the best proven interventions must be evaluated continually through research for their safety, effectiveness, efficiency, accessibility and quality.

7. Medical research is subject to ethical standards that promote and ensure respect for all human subjects and protect their health and rights.

8. While the primary purpose of medical research is to generate new knowledge, this goal can never take precedence over the rights and interests of individual research subjects.

9. It is the duty of physicians who are involved in medical research to protect the life, health, dignity, integrity, right to self-determination, privacy, and confidentiality of personal information of research subjects. The responsibility for the protection of research subjects must always rest with the physician or other health care professionals and never with the research subjects, even though they have given consent.

10. Physicians must consider the ethical, legal and regulatory norms and standards for research involving human subjects in their own countries as well as applicable international norms and standards. No national or international ethical, legal or regulatory requirement should reduce or eliminate any of the protections for research subjects set forth in this Declaration.

11. Medical research should be conducted in a manner that minimises possible harm to the environment.

12. Medical research involving human subjects must be conducted only by individuals with the appropriate ethics and scientific education, training and qualifications. Research on patients or healthy volunteers requires the supervision of a competent and appropriately qualified physician or other health care professional.

13. Groups that are underrepresented in medical research should be provided appropriate access to participation in research.

14. Physicians who combine medical research with medical care should involve their patients in research only to the extent that this is justified by its potential preventive, diagnostic or therapeutic value and if the physician has good reason to believe that participation in the research study will not adversely affect the health of the patients who serve as research subjects.

15. Appropriate compensation and treatment for subjects who are harmed as a result of participating in research must be ensured.

Risks, Burdens and Benefits

16. In medical practice and in medical research, most interventions involve risks and burdens.

Medical research involving human subjects may only be conducted if the importance of the objective outweighs the risks and burdens to the research subjects.

17. All medical research involving human subjects must be preceded by careful assessment of predictable risks and burdens to the individuals and groups involved in the research in comparison with foreseeable benefits to them and to other individuals or groups affected by the condition under investigation.

Measures to minimise the risks must be implemented. The risks must be continuously monitored, assessed and documented by the researcher.

18. Physicians may not be involved in a research study involving human subjects unless they are confident that the risks have been adequately assessed and can be satisfactorily managed.

STRICTLY CONFIDENTIAL: Property of Hospital Universitario de la Princesa.
It is not allowed the dissemination or replication out of terms of this proposal

When the risks are found to outweigh the potential benefits or when there is conclusive proof of definitive outcomes, physicians must assess whether to continue, modify or immediately stop the study.

Vulnerable Groups and Individuals

19. Some groups and individuals are particularly vulnerable and may have an increased likelihood of being wronged or of incurring additional harm.

All vulnerable groups and individuals should receive specifically considered protection.

20. Medical research with a vulnerable group is only justified if the research is responsive to the health needs or priorities of this group and the research cannot be carried out in a non-vulnerable group. In addition, this group should stand to benefit from the knowledge, practices or interventions that result from the research.

Scientific Requirements and Research Protocols

21. Medical research involving human subjects must conform to generally accepted scientific principles, be based on a thorough knowledge of the scientific literature, other relevant sources of information, and adequate laboratory and, as appropriate, animal experimentation. The welfare of animals used for research must be respected.

22. The design and performance of each research study involving human subjects must be clearly described and justified in a research protocol.

The protocol should contain a statement of the ethical considerations involved and should indicate how the principles in this Declaration have been addressed. The protocol should include information regarding funding, sponsors, institutional affiliations, potential conflicts of interest, incentives for subjects and information regarding provisions for treating and/or compensating subjects who are harmed as a consequence of participation in the research study.

In clinical trials, the protocol must also describe appropriate arrangements for post-trial provisions.

Research Ethics Committees

23. The research protocol must be submitted for consideration, comment, guidance and approval to the concerned research ethics committee before the study begins. This committee must be transparent in its functioning, must be independent of the researcher, the sponsor and any other undue influence and must be duly qualified. It must take into consideration the laws and regulations of the country or countries in which the research is to be performed as well as applicable international norms and standards but these must not be allowed to reduce or eliminate any of the protections for research subjects set forth in this Declaration.

The committee must have the right to monitor ongoing studies. The researcher must provide monitoring information to the committee, especially information about any serious adverse events. No amendment to the protocol may be made without consideration and approval by the committee. After the end of the study, the researchers must submit a final report to the committee containing a summary of the study's findings and conclusions.

Privacy and Confidentiality

STRICTLY CONFIDENTIAL: Property of Hospital Universitario de la Princesa.
It is not allowed the dissemination or replication out of terms of this proposal

24. Every precaution must be taken to protect the privacy of research subjects and the confidentiality of their personal information.

Informed Consent

25. Participation by individuals capable of giving informed consent as subjects in medical research must be voluntary. Although it may be appropriate to consult family members or community leaders, no individual capable of giving informed consent may be enrolled in a research study unless he or she freely agrees.

26. In medical research involving human subjects capable of giving informed consent, each potential subject must be adequately informed of the aims, methods, sources of funding, any possible conflicts of interest, institutional affiliations of the researcher, the anticipated benefits and potential risks of the study and the discomfort it may entail, post-study provisions and any other relevant aspects of the study. The potential subject must be informed of the right to refuse to participate in the study or to withdraw consent to participate at any time without reprisal. Special attention should be given to the specific information needs of individual potential subjects as well as to the methods used to deliver the information.

After ensuring that the potential subject has understood the information, the physician or another appropriately qualified individual must then seek the potential subject's freely-given informed consent, preferably in writing. If the consent cannot be expressed in writing, the non-written consent must be formally documented and witnessed.

All medical research subjects should be given the option of being informed about the general outcome and results of the study.

27. When seeking informed consent for participation in a research study the physician must be particularly cautious if the potential subject is in a dependent relationship with the physician or may consent under duress. In such situations the informed consent must be sought by an appropriately qualified individual who is completely independent of this relationship.

28. For a potential research subject who is incapable of giving informed consent, the physician must seek informed consent from the legally authorised representative. These individuals must not be included in a research study that has no likelihood of benefit for them unless it is intended to promote the health of the group represented by the potential subject, the research cannot instead be performed with persons capable of providing informed consent, and the research entails only minimal risk and minimal burden.

29. When a potential research subject who is deemed incapable of giving informed consent is able to give assent to decisions about participation in research, the physician must seek that assent in addition to the consent of the legally authorised representative. The potential subject's dissent should be respected.

30. Research involving subjects who are physically or mentally incapable of giving consent, for example, unconscious patients, may be done only if the physical or mental condition that prevents giving informed consent is a necessary characteristic of the research group. In such circumstances the physician must seek informed consent from the legally authorised representative. If no such representative is available and if the research cannot be delayed, the study may proceed without informed consent provided that the specific reasons for involving subjects with a condition that renders them unable to give informed consent have been stated in the research protocol and the study has been approved by a research ethics committee. Consent to remain in the research must be obtained as soon as possible from the subject or a legally authorised representative.

STRICTLY CONFIDENTIAL: Property of Hospital Universitario de la Princesa.
It is not allowed the dissemination or replication out of terms of this proposal

31. The physician must fully inform the patient which aspects of their care are related to the research. The refusal of a patient to participate in a study or the patient's decision to withdraw from the study must never adversely affect the patient-physician relationship.

32. For medical research using identifiable human material or data, such as research on material or data contained in biobanks or similar repositories, physicians must seek informed consent for its collection, storage and/or reuse. There may be exceptional situations where consent would be impossible or impracticable to obtain for such research. In such situations the research may be done only after consideration and approval of a research ethics committee.

Use of Placebo

33. The benefits, risks, burdens and effectiveness of a new intervention must be tested against those of the best proven intervention(s), except in the following circumstances:

Where no proven intervention exists, the use of placebo, or no intervention, is acceptable; or

Where for compelling and scientifically sound methodological reasons the use of any intervention less effective than the best proven one, the use of placebo, or no intervention is necessary to determine the efficacy or safety of an intervention

and the patients who receive any intervention less effective than the best proven one, placebo, or no intervention will not be subject to additional risks of serious or irreversible harm as a result of not receiving the best proven intervention.

Extreme care must be taken to avoid abuse of this option.

Post-Trial Provisions

34. In advance of a clinical trial, sponsors, researchers and host country governments should make provisions for post-trial access for all participants who still need an intervention identified as beneficial in the trial. This information must also be disclosed to participants during the informed consent process.

Research Registration and Publication and Dissemination of Results

35. Every research study involving human subjects must be registered in a publicly accessible database before recruitment of the first subject.

36. Researchers, authors, sponsors, editors and publishers all have ethical obligations with regard to the publication and dissemination of the results of research. Researchers have a duty to make publicly available the results of their research on human subjects and are accountable for the completeness and accuracy of their reports. All parties should adhere to accepted guidelines for ethical reporting. Negative and inconclusive as well as positive results must be published or otherwise made publicly available. Sources of funding, institutional affiliations and conflicts of interest must be declared in the publication. Reports of research not in accordance with the principles of this Declaration should not be accepted for publication.

Unproven Interventions in Clinical Practice

37. In the treatment of an individual patient, where proven interventions do not exist or other known interventions have been ineffective, the physician, after seeking expert advice, with informed consent from the patient or a legally authorised representative, may use an unproven intervention if in

the physician's judgement it offers hope of saving life, re-establishing health or alleviating suffering. This intervention should subsequently be made the object of research, designed to evaluate its safety and efficacy. In all cases, new information must be recorded and, where appropriate, made publicly available

APPENDIX B: Serious and Unexpected Adverse Reactions Notification Form (SUARs Form) (Spanish).

INSTRUCCIONES GENERALES

1. Este formulario se utilizará solamente para comunicar las sospechas de reacciones adversas (RA) graves e inesperadas que ocurran con medicamentos en investigación.

2. Las sospechas de RA mortales o que entrañen riesgo vital (aquellas que de no haber mediado una intervención terapéutica inmediata hubieran supuesto la muerte del paciente) se comunicarán en el plazo máximo de 7 días naturales; si no se dispusiera de toda la información, ésta podrá completarse en el plazo adicional de 8 días. Las demás sospechas de RA graves e inesperadas se comunicarán en el plazo máximo de 15 días.

3. Cuando el espacio disponible sea insuficiente, se añadirá una hoja de información adicional, correctamente identificada con el nombre del sponsor y el número asignado a la notificación. En dicha información podrá hacerse constar la evaluación de la causalidad realizada por el técnico que informa.

INSTRUCCIONES ESPECÍFICAS

Ø. El código de protocolo es el asignado por el sponsor para identificar el ensayo. El número de notificación del sponsor es el que éste utiliza para su archivo. Cuando se trate de información de seguimiento se utilizará el mismo número o bien, si se modifica, se indicará el número de la notificación inicial. Se dejará sin rellenar el espacio "Nº de notificación" que aparece sombreado.

2. La edad se pondrá en años, meses, semanas o días según convenga, pero siempre indicándolo. Si no se conoce con precisión la edad debe referirse, al menos, el grupo de edad al que pertenece (p. ej.: lactante, niño, adolescente, adulto, anciano).

7. Se describirá la RA en forma completa, indicando la fecha de finalización de la misma e incluyendo los resultados de las exploraciones complementarias o pruebas de laboratorio que se consideren de interés. A esta notificación podrán acompañarse cuantos informes se estimen convenientes para la adecuada interpretación del cuadro clínico sospechoso de ser una reacción adversa.

8-13. Las categorías no son mutuamente excluyentes. La asistencia en un Servicio de Urgencias de un Hospital inferior a 24 horas, no se considerará hospitalización.

14. Los medicamentos en investigación se identificarán a ser posible por su nombre genérico (DOE o DCI), indicando cuando esté disponible el nombre comercial, o en su defecto, por el nombre propuesto o código de laboratorio para el producto.

15. En caso de que la administración no sea diaria se intentará describirla con alguna de las siguientes posibilidades: cíclica, semanal, mensual, anual o número de veces que se ha utilizado (poniendo en este caso la dosis de cada toma, no la total).

17. Se hará constar el proceso patológico del paciente al que va destinado el producto en investigación, o bien "voluntario sano" en caso de tratarse de tal.

STRICTLY CONFIDENTIAL: Property of Hospital Universitario de la Princesa.
It is not allowed the dissemination or replication out of terms of this proposal

19. Se hará constar la duración del tratamiento hasta el inicio de la reacción adversa.

22. Se indicará explícitamente si no se han tomado fármacos concomitantes. En el caso de considerar sospechoso alguno o algunos de los fármacos concomitantes se marcarán con un asterisco (ej: * AMOXICILINA). Se excluirán los medicamentos utilizados para tratar la reacción adversa.

Anexo D: Formulario de notificación de reacción adversa grave e inesperada (RAGI) ocurrida en España

NOTIFICACIÓN DE SOSPECHA DE REACCIÓN ADVERSA PARA MEDICAMENTOS EN INVESTIGACIÓN	CÓDIGO DE PROTOCOLO (sponsor) .... Nº EUDRACT/Nº Protocolo AEMPS	Nº NOTIFICACION (Sponsor)
Notificación realizada a Eudravigilance ☐ SI ☐ NO	PACIENTE Nº	Nº NOTIFICACION.............

INFORMACIÓN SOBRE LA REACCIÓN ADVERSA

1a. PAÍS	2. FECHA DE NACIMIENTO			2a. EDAD	3. SEXO	3a. PESO	3b. TALLA	4-6. FECHA DE INICIO DE LA REACCIÓN		
**ESP**	DÍA	MES	AÑO		☐ HOMBRE<br>☐ MUJER			DÍA	MES	AÑO

7. DESCRIPCIÓN DE LA REACCIÓN ADVERSA (Incluyendo resultados relevantes de exploración o de laboratorio, y la fecha de finalización, si procede).	8-13b. CRITERIOS DE GRAVEDAD/DESENLACE<br>**Gravedad**<br>☐ FALLECIMIENTO<br>☐ LA VIDA DEL PACIENTE HA ESTADO EN PELIGRO<br>☐ HOSPITALIZACIÓN<br>☐ PROLONGACIÓN HOSPITALIZACIÓN<br>☐ INCAPACIDAD PERMANENTE O SIGNIFICATIVA<br>☐ RA CLÍNICAMENTE RELEVANTE<br>**Desenlace**<br>☐ PERSISTENCIA DE LA RA<br>☐ RECUPERACIÓN SIN SECUELAS<br>☐ RECUPERACIÓN CON SECUELAS<br>☐ DESCONOCIDO

II. INFORMACIÓN DEL MEDICAMENTO EN INVESTIGACIÓN

14. MEDICAMENTO SOSPECHOSO	15. DOSIS DIARIA	16. VÍA	17. ENFERMEDAD EN ESTUDIO	18. FECHAS DE INICIO	FINAL	19. DURACIÓN DEL TRATAMIENTO

20. ¿REMITIÓ LA REACCIÓN AL SUSPENDER LA MEDICACIÓN?	20a. ¿REMITIÓ LA REACCIÓN AL REDUCIR LA DOSIS?	21. ¿REAPARECIÓ LA REACCIÓN AL ADMINISTRAR DE NUEVO LA MEDICACIÓN?
☐ SI ☐ NO ☐ NO PROCEDE	☐ SI ☐ NO ☐ NO PROCEDE	☐ SI ☐ NO ☐ NO PROCEDE

III. MEDICAMENTOS CONCOMITANTES E HISTORIA CLÍNICA

22. MEDICAMENTOS CONCOMITANTES (Márquese con un asterisco el o los medicamentos sospechosos)	22a. DOSIS DIARIA	22b. VÍA	22c. FECHAS DE INICIO	FINAL	22d. MOTIVO DE LA PRESCRIPCIÓN
23. DATOS IMPORTANTES DE LA HISTORIA CLÍNICA (ej. diagnósticos, alergias, embarazos, etc.)					

IV. INFORMACIÓN SOBRE SPONSOR E INVESTIGADOR

STRICTLY CONFIDENTIAL: Property of Hospital Universitario de la Princesa.
It is not allowed the dissemination or replication out of terms of this proposal

24a. NOMBRE Y DIRECCIÓN DEL SPONSOR		24b. NOMBRE Y DIRECCIÓN DEL INVESTIGADOR
24c. CÓDIGO DE LABORATORIO<br>(Nº AEMPS)	25a. TIPO DE INFORME<br><br>☐ INICIAL<br>☐ SEGUIMIENTO	24d. TÉCNICO DEL SPONSOR QUE INFORMA<br>NOMBRE:<br>TELÉFONO:<br>FIRMA:
24e. FECHA DEL INFORME	24f. FECHA DE ENTRADA AEMPS	25b. SE ADJUNTA INFORME COMPLEMENTARIO

APPENDIX C: authorization from the Spanish Agency for Medicines and Healthcare Products (AEMPS) and the favorable opinion of a Committee on Ethics of Research with Medicines.

GOBIERNO DE ESPAÑA — MINISTERIO DE SANIDAD — agencia española de medicamentos y productos sanitarios

DEPARTAMENTO DE MEDICAMENTOS DE USO HUMANO
Área de Ensayos Clínicos

Referencia: MUH/CLIN/EC

ASUNTO: RESOLUCIÓN DE LA SOLICITUD DE AUTORIZACIÓN DE UN ENSAYO CLÍNICO

DESTINATARIO: Rosario García de Vicuña
Diego de León 62
28006 Madrid (España)

DATOS DE LA SOLICITUD

Solicitud de autorizacion del Ensayo clinico Nº EudraCT **2020-001634-36** y título **Estudio piloto abierto aleatorizado para evaluar la eficacia de sarilumab subcutáneo en pacientes con infección por COVID-19 moderada-grave..**

Promotor: Rosario García de Vicuña
Diego de León 62
28006 Madrid (España)

Fecha de solicitud válida: 10/04/2020

Una vez evaluada la solicitud de autorización de ensayo clínico previamente indicada, se considera que cumple con los requisitos indicados en el Real Decreto 1090/2015, de 4 de diciembre, por el que se regulan los ensayos clínicos con medicamentos, los Comités de Ética de la Investigación con medicamentos y el Registro Español de Estudios Clínicos y demás legislación aplicable*.

Por todo lo anteriormente expuesto la Directora de la Agencia de Medicamentos y Productos Sanitarios en el ejercicio de sus competencias **RESUELVE:**

AUTORIZAR el ensayo clínico solicitado.

COMENTARIOS A LA AUTORIZACIÓN:

En aras de la salud pública es imprescindible trasladar lo más rápidamente posible a la práctica clínica la evidencia científica que generen las investigaciones sobre el COVID-19.

* Texto refundido de la Ley de Garantías y Uso Racional de los medicamentos y productos sanitarios, aprobado por Real Decreto Legislativo 1/2015, de 24 de julio.
Real Decreto 1275/2011, de 16 de septiembre, por el que se crea la Agencia estatal "Agencia Española de Medicamentos y Productos Sanitarios y se aprueba su Estatuto".

Agencia Española de Medicamentos y Productos Sanitarios — **Localizador: C X 8 J 8 E Q 5 A C**
Fecha de la firma: 08/04/2020
Puede comprobar la autenticidad del documento en la sede de la AEMPS: https://sede.aemps.gob.es

CORREO ELECTRÓNICO
smhaem@aemps.es — Página 1 de 2 — C/ CAMPEZO, 1 - EDIFICIO 8, 28022 MADRID, Tel.: 918225073, Fax: 918225043

Por ello, le rogamos encarecidamente que comunique a la AEMPS los resultados preliminares de este estudio en cuanto estén disponibles, independientemente de su ulterior publicación en revistas científicas.

Contra esta Resolución, que pone fin a la vía administrativa, puede interponerse potestativamente Recurso de Reposición ante el/la Director/a de la Agencia Española de Medicamentos y Productos Sanitarios en el plazo de un mes, conforme a lo dispuesto en los artículos 123 y 124 de la Ley 39/2015, de 1 de octubre, del Procedimiento Administrativo Común de las Administraciones Públicas, o interponerse Recurso Contencioso-Administrativo ante el Juzgado Central de lo Contencioso-Administrativo de Madrid, en el plazo de dos meses a contar desde el día siguiente a la recepción de la presente notificación, conforme a lo dispuesto en la Ley Reguladora de la Jurisdicción Contencioso-Administrativa de 13 de julio de 1998, y sin perjuicio de cualquier otro recurso que pudiera interponerse.

DIRECTORA DE LA AGENCIA ESPAÑOLA DE MEDICAMENTOS Y PRODUCTOS SANITARIOS

agencia española de medicamentos y productos sanitarios

Fdo. Mª Jesús Lamas Díaz

Agencia Española de Medicamentos y Productos Sanitarios — **Localizador: C X 8 J 8 E Q 5 A C**
Fecha de la firma: 08/04/2020
Puede comprobar la autenticidad del documento en la sede de la AEMPS: https://sede.aemps.gob.es

CORREO ELECTRÓNICO
smhaem@aemps.es — Página 2 de 2 — C/ CAMPEZO, 1 - EDIFICIO 8, 28022 MADRID, Tel.: 918225073, Fax: 918225043

SaludMadrid Hospital Universitario de La Princesa

Comunidad de Madrid

DICTAMEN DEL COMITÉ DE ÉTICA DE LA INVESTIGACIÓN CON MEDICAMENTOS

Dña. Mª del Mar Ortega Gómez Secretaria del **COMITÉ ÉTICO DE INVESTIGACIÓN CLÍNICA CON MEDICAMENTOS del Hospital Universitario de la Princesa**

CERTIFICA

Que este Comité ha evaluado la siguiente propuesta de ensayo clínico de bajo nivel de intervención:

CÓDIGO: SARCOVID
NÚMERO EudraCT: 2020-001634-36
TÍTULO: Estudio piloto abierto aleatorizado para evaluar la eficacia de sarilumab subcutáneo en pacientes con infección por COVID-19 moderada-grave (versión 2-corr, 6-4-20).
PROMOTOR: Rosario García de Vicuña
PROTOCOLO: Versión 2-corr: 06-04-2020
HIP/CI GENERAL: Versión 2-corr: 06-04-2020

Que este Comité ha realizado la evaluación de la parte I de la solicitud de autorización del ensayo y ha transmitido a la Agencia Española de medicamentos su opinión final sobre la parte I.

Que este Comité ha realizado la evaluación de la parte II de la solicitud de autorización del ensayo, de acuerdo con lo previsto en el Real Decreto 1090/2015 y en el artículo 7 del reglamento (UE) 536/2014 y considera que:

- El procedimiento para obtener el consentimiento informado (incluyendo las hojas de información al sujeto de ensayo y consentimientos informados mencionados en el encabezamiento), y el plan de reclutamiento de sujetos previsto son adecuados y cumplen con los requisitos para la obtención del consentimiento informado previstos en el capítulo II del Real Decreto 1090/2015.
- Las compensaciones previstas a los participantes son adecuadas, así como las previsiones de indemnización por daños y perjuicios que pueda sufrir el participante.
- El procedimiento previsto para el manejo de datos personales es adecuado.
- El uso futuro de las muestras biológicas obtenidas durante el ensayo se adecua a lo previsto en el Real Decreto 1716/2011.
- Para la realización del ensayo se consideran adecuados los centros e investigadores previstos en el anexo II a este dictamen, teniendo en cuenta las declaraciones de idoneidad emitidas por el promotor y por los responsables de las instituciones correspondientes.
- Que este Comité decidió emitir **DICTAMEN FAVORABLE** el día **09-04-2020 (Acta CEIm nº 08/20)**

CEIm Hospital Universitario La Princesa. C/ Diego de León, 62. 28006. Madrid
Tel. 91 520 24 76/Fax: 91 520 25 60. ceim.hlpr@salud.madrid.org

SaludMadrid Hospital Universitario de La Princesa

Comunidad de Madrid

- Que en dicha reunión se cumplieron los requisitos establecidos en la legislación vigente –Real Decreto 1090/2015 – para que la decisión del citado CEIm sea válida.
- Que el CEIm del Hospital Universitario de la Princesa, tanto en su composición como en sus procedimientos, cumple con las normas de BPC (CPMP/ICH/135/95) y con la legislación vigente que regula su funcionamiento, y que la composición del CEIm del Hospital Universitario de la Princesa es la indicada en el anexo I, teniendo en cuenta que en el caso de que algún miembro participe en el ensayo o declare algún conflicto de interés no habrá participado en la evaluación ni en el dictamen de la solicitud de autorización del ensayo clínico.

Lo que firmo en Madrid, a 9 de abril de 2020

ORTEGA GOMEZ MARIA DEL MAR - 52114122D

Firmado digitalmente por ORTEGA GOMEZ MARIA DEL MAR - 52114122D
Nombre de reconocimiento (DN): c=ES, serialNumber=IDCES-52114122D, givenName=MARIA DEL MAR, sn=ORTEGA GOMEZ, cn=ORTEGA GOMEZ MARIA DEL MAR - 52114122D
Fecha: 2020.04.09 11:25:09 +02'00'

Fdo.: Dra. Mª del Mar Ortega Gómez
SECRETARIA DEL CEIm

STRICTLY CONFIDENTIAL: Property of Hospital Universitario de la Princesa.
It is not allowed the dissemination or replication out of terms of this proposal
